# Seasonal drought in North America’s sagebrush biome structures dynamic mesic resources for sage‐grouse

**DOI:** 10.1002/ece3.4614

**Published:** 2018-12-11

**Authors:** J. Patrick Donnelly, Brady W. Allred, Daniel Perret, Nicholas L. Silverman, Jason D. Tack, Victoria J. Dreitz, Jeremy D. Maestas, David E. Naugle

**Affiliations:** ^1^ Intermountain West Joint Venture Missoula Montana; ^2^ United States Fish and Wildlife Service Missoula Montana; ^3^ WA Franke College of Forestry and Conservation University of Montana Missoula Montana; ^4^ Department of Ecology and Evolutionary Biology Brown University Providence Rhode Island; ^5^ Avian Science Center and Wildlife Biology Program University of Montana Missoula Montana; ^6^ Natural Resources Conservation Service Portland Oregon; ^7^ Natural Resources Conservation Service—Sage Grouse Initiative Missoula Montana

**Keywords:** drought, Great Basin, Great Plains, net primary productivity, precipitation, Rocky Mountains, sagebrush biome, sage‐grouse

## Abstract

The North American semi‐arid sagebrush, *Artemisia* spp., biome exhibits considerable climatic complexity driving dynamic spatiotemporal shifts in primary productivity. Greater and Gunnison sage‐grouse, *Centrocercus urophasianus* and *C. minimus*, are adapted to patterns of resource intermittence and rely on stable adult survival supplemented by occasional recruitment pulses when climatic conditions are favorable. Predictions of intensifying water scarcity raise concerns over new demographic bottlenecks impacting sage‐grouse populations in drought‐sensitive landscapes. We estimate biome‐wide mesic resource productivity from 1984 to 2016 using remote sensing to identify patterns of food availability influencing selective pressures on sage‐grouse. We linked productivity to abiotic factors to examine effects of seasonal drought across time, space, and land tenure, with findings partitioned along gradients of ecosystem water balance within Great Basin, Rocky Mountains and Great Plains regions. Precipitation was the driver of mesic resource abundance explaining ≥70% of variance in drought‐limited vegetative productivity. Spatiotemporal shifts in mesic abundance were apparent given biome‐wide climatic trends that reduced precipitation below three‐quarters of normal in 20% of years. Drought sensitivity structured grouse populations wherein landscapes with the greatest uncertainty in mesic abundance and distribution supported the fewest grouse. Privately owned lands encompassed 40% of sage‐grouse range, but contained a disproportional 68% of mesic resources. Regional drought sensitivity identified herein acted as ecological minimums to influence differences in landscape carrying capacity across sage‐grouse range. Our model depictions likely reflect a new normal in water scarcity that could compound impacts of demographic bottlenecks in Great Basin and Great Plains. We conclude that long‐term population maintenance depends on a diversity of drought resistant mesic resources that offset climate driven variability in vegetative productivity. We recommend a holistic public–private lands approach to mesic restoration to offset a deepening risk of water scarcity.

## 1 INTRODUCTION

Water input is one of the most dynamic determinants of terrestrial productivity in arid and semi‐arid regions and is fundamental to biological processes responsible for ecosystem function. Drought‐prone ecosystems account for 40% of terrestrial land surface globally and support upwards of 2 billion people (Gilbert, 2011). These regions are defined by relatively low mean annual precipitation rates and excessive evapotranspirative demands that directly influence primary production (Le Houérou, Bingham, & Skerbek, 1988; Noy‐Meir, 1973). Annual and intra‐annual variability in precipitation is typical with distinct dry seasons and unpredictable prolonged droughts that lead to frequent periods of water scarcity (Schlesinger et al., 1990). Human population growth, combined with shifts in climatic conditions, is likely to increase pressure on these ecosystems and further strain already limiting water resources (MEAB, 2005).

Drought is a main driver of reduction in aboveground net primary production (NPP; Webb, Lauenroth, Szarek, & Kinerson, 1983). In arid and semi‐arid ecosystems, seasonal drought often limits resource availability, serving as an ecological minimum or bottleneck, thereby reducing the abundance and distribution of associated wildlife populations (Bolger, Patten, & Bostock, 2005). Nonlinear landscape response during these periods can generate distinct spatial patterns of vegetative productivity associated with contrasting soil moisture dynamics and floral communities that structure geographic distribution of resources (Knapp & Smith, 2001). Efficiencies in arid lands conservation rely on an ability to predict landscape response to offset water scarcity, yet our understanding of drought to structure spatiotemporal intermittence of primary production remains limited (Vicente‐Serrano et al., 2013). Misalignment of conservation actions can impede ecosystem benefits needed to counteract predicted climatic fluctuations that have the potential to operate as new and powerful ecological constraints to biodiversity in semi‐arid landscapes (Maron, McAlpine, & Watson, 2015).

The greater and Gunnison sage‐grouse (*Centrocercus urophasianus* and *C. minimus*; hereafter “sage‐grouse”) are gallinaceous birds and indicator species of the semi‐arid sagebrush (*Artemisia* spp.) biome of western North America (Rowland, Wisdom, Suring, & Meinke, 2006; Schroeder et al., 2004). Sage‐grouse are emblematic of water scarcity because they exhibit a bottleneck in reproductive cost associated with nutritional stress that aligns with periods of seasonal drought in late summer (Blomberg, Sedinger, Nonne, & Atamian, 2013). Seasonal drying and senescence of herbaceous vegetation induce sage‐grouse to seek out few remaining productive sites associated with high‐value foraging habitat for chick growth and survival. Sites include wet meadows, riparian corridors, drought resistant rangelands and irrigated alfalfa, hereafter “mesic resources” (Connelly, Rinkes, & Braun, 2011). Sage‐grouse are representative of spatial patterns in drought‐induced ecological minimums, as productive mesic sites in late summer provide an important, but limiting food resource that structure sage‐grouse abundance and distribution within broader landscapes (Donnelly, Naugle, Hagen, & Maestas, 2016). Significant temperature increases across western North America in recent decades (Melillo, Richmond, & Yohe, 2014) now threaten availability of mesic resources that have foreshadowed growing concerns of deepening drought and its effect on sage‐grouse populations (Gibson, Blomberg, Atamian, & Sedinger, 2017; Guttery et al., 2013).

Restoration and maintenance of mesic resource productivity in sagebrush landscapes must rely on geographically specific conservation strategies adapted to address regional drought sensitivities. The sagebrush biome exhibits considerable complexity in precipitation magnitude, timing, and variation (Rajagopalan & Lall, 1998). Spatially dynamic patterns of resource availability driven by these processes produce disparities in habitat quality and wildlife fitness potential within landscapes (Coates et al., 2016; Pastor, Moen, & Cohen, 1997). Wide‐ranging species like sage‐grouse may experience regional variability in selective pressures associated with nonlinear landscape response to drought as soil moisture is a primary driver of seasonally important food resources associated with mesic sites (e.g., forbs and macro invertebrates, Wenninger & Inouye, 2008). Further complicating water scarcity are geographical shifts in proportional public and private land ownership (Donnelly et al., 2016) linked to regionally pervasive land‐use practices that can inhibit or promote drought effects.

Changes to large‐scale ecosystem dynamics increasingly require more than just field surveys to understand, monitor, and report on their effects (Marvin et al., 2016). To better quantify landscape sensitivity to seasonal drought and its influence in structuring mesic resources for sage‐grouse, we leveraged over 15,000 satellite images to produce a spatiotemporal dataset that tracked annual vegetative productivity patterns across the sagebrush biome from 1984 to 2016. Productivity data were combined with land tenure information to evaluate proportional mesic resource abundance by ownership. The new dataset introduces geographic scale and perspective to ecological drought and its relationship to sage‐grouse that to this point remain unexplored. Results provide a framework that for the first time links local evidence‐based studies to rangewide drought effects influencing demographic constraints in sage‐grouse populations (Blomberg, Sedinger, Atamian, & Nonne, 2012; Blomberg, Sedinger, Gibson, Coates, & Casazza, 2014; Gibson et al., 2017; Guttery et al., 2013). Study outcomes deliver new insight to support development of regionally specific conservation strategies necessary to offset drought‐induced bottlenecks impacting sage‐grouse and other drought sensitive wildlife in sagebrush ecosystems.

## 2 MATERIALS AND METHODS

### 2.1 Study site

**Figure 1:**
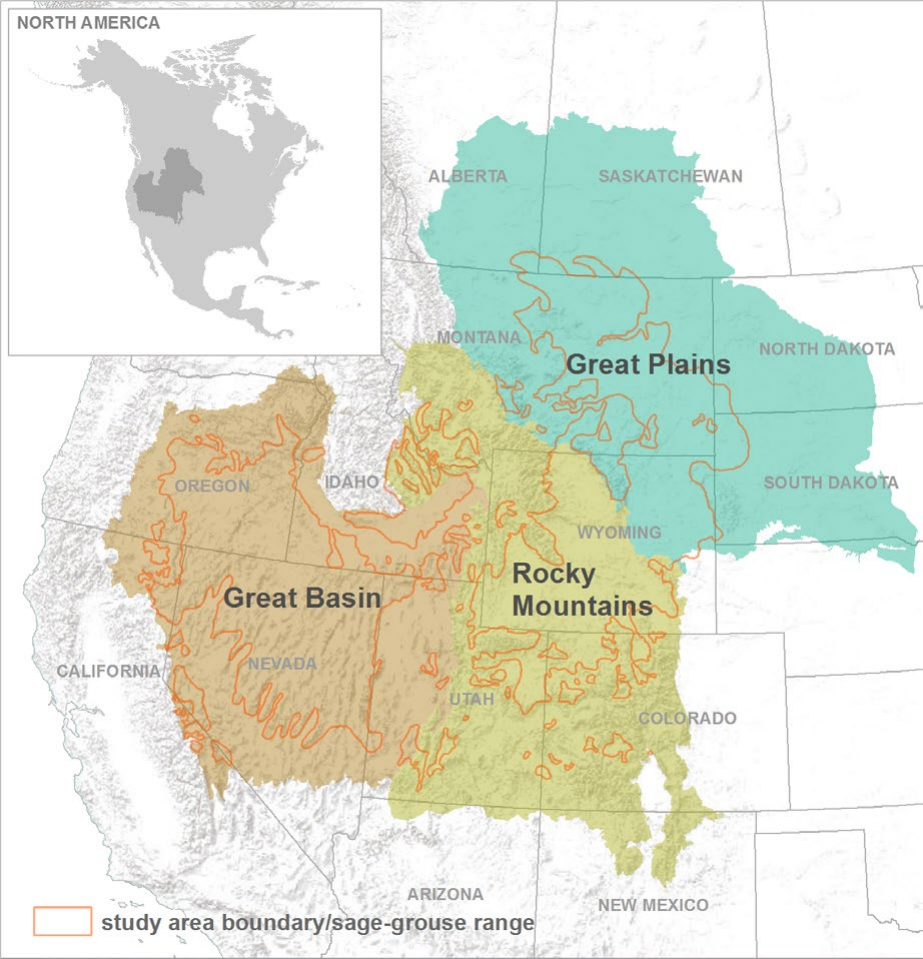
Study area delineated using sage‐grouse range as a proxy to define sagebrush biome. The study area was divided into three areas (Great Basin, Rocky Mountains, and Great Plains) along gradients of broader ecosystem water balance as shown

We used current sage‐grouse range (>74 million ha, Schroeder et al., 2004) to delineate the sagebrush biome and define the study area boundary (Figure 1). Sage‐grouse distribution provided a requisite for large and unfragmented sagebrush landscapes as >90% of birds occur in areas of >40% sagebrush land‐cover (Knick, Hanser, & Preston, 2013). Sage‐grouse range was divided into three regions (Great Basin, Rocky Mountains, and Great Plains) along gradients of ecosystem water balance using climatic differences associated with temporal overlap of seasonal precipitation and evapotranspiration (Sala, Lauenroth, & Golluscio, 1997, Figure 1). Alignment of these processes correlates to differences in soil water availability (Lauenroth, Schlaepfer, & Bradford, 2014). Summer precipitation occurring when temperature and plant growth are high and potential evapotranspiration (PET; Black, 2007) is greatest supports pulse soil water dynamics that minimize deep soil water storage (Sala, Lauenroth, & Parton, 1992). The opposite occurs when overlap is poor and precipitation comes during cold periods when PET is lowest and potential for deep soil water storage is maximized.

We estimated gradients of pulse and deep soil water potential by calculating correlation coefficients of daily precipitation and PET (Schlaepfer, Lauenroth, & Bradford, 2012). Estimates were calculated within water years (from 01 October to 30 September) and fit to a continuous grid (4 × 4 km) using day of year precipitation and day of year PET means for the study period (1984 to 2016) obtained from Gridded Surface Meteorological Dataset (GRIDMET; Abatzoglou, 2013). Results were scaled from negative one to one and interpreted to be more predictive of summer precipitation pulse soil water potential as positive values increased toward one. Negative values decreasing to negative one were considered more predictive of cold season precipitation and deep soil water potential, with values near zero predictive of both pulse and deep soil water characteristics. We averaged coefficient values within North American level III ecoregions (https://www.epa.gov/eco-research/ecoregions) by assigning the mean of intersecting grid cells to each ecoregion. Ecoregions were aggregated geographically using a k‐means clustering analysis of their correlation coefficient mean and intersected with current sage‐grouse range to form the Great Basin, Rocky Mountains, and Great Plains regions (Figure 1).

Portions of sage‑grouse range in Canada (<1% of study area) were excluded from correlation coefficient calculations because continuous estimates of PET were unavailable. Sagebrush steppe landscapes in eastern Washington, United States, were omitted from our analysis due to the area's high rate of fragmentation and agricultural influences that were uncharacteristic of the remainder of sage‐grouse range (Shirk, Schroeder, Robb, & Cushman, 2017).

### 2.2 Modeling drought limited primary production

In semi‐arid ecosystems, aboveground NPP is controlled by patterns of soil water availability (Noy‐Meir, 1973). We examined this concept by delineating spatiotemporal patterns of mesic resource productivity as a proxy to soil moisture derived from Landsat 4, 5, and 8 satellite imagery. Measurements were based on normalized difference vegetation indices (NDVI) which quantify photosynthetic activity and correlate closely to fluctuations in NPP (Box, Holben, & Kalb, 1989; Pettorelli et al., 2005). Satellite images (*n* = 5,180) were used to conduct annual monitoring during a 33‐year span (1984–2011 and 2013–2016) to account for climate driven variation in landscape condition (Loik, Breshears, Lauenroth, & Belnap, 2004). Poor‐quality Landsat 7 imagery prevented monitoring in 2012. All images were calibrated across sensors and corrected for atmospheric effects and illumination/viewing geometry (Masek et al., 2006; Vermote, Justice, Claverie, & Franch, 2016). Pixels containing surface anomalies (i.e., cloud, cloud shadow, snow, and water) were removed using the Landsat CFMask band. Images were acquired 15 July to 30 September to coincide with high evapotranspiration demand in sagebrush ecosystems when mesic food resource availability for sage‐grouse is restricted (Connelly, Rinkes, et al., 2011) and patterns of drought‐induced vegetative productivity are evident in semi‐arid systems (Vicente‐Serrano et al., 2013).

To delineate spatial patterns of mesic resources, we generated a raster image (30x30 m pixel) of maximum NDVI values selected from 32 years of overlapping Landsat images (*n* = 15,180) used in our analysis. Individual pixels were representative of the highest primary productivity measured 15 July to 30 September, 1984 to 2016. Results were representative of maximum landscape productivity potential during seasonal drought. We applied an object‐based segmentation algorithm to this image using program eCognition 9.2 (https://www.ecognition.com) that clustered pixels into polygons representative of underlying landscape features. The resulting polygon layer was used in our model as a non‐uniform sampling grid (hereafter “mesic grid”) to estimate availability and spatial distribution of vegetative productivity. Only polygons containing mean and maximum NDVI pixel values indicative of higher primary productivity (≥0.3) were retained. We considered this NDVI value as the threshold separating non‐productive sites from productive and drought resilient sites with high value for foraging sage‐grouse (Donnelly et al., 2016; Weier & Herring, 2000). NDVI polygon values under this threshold were considered to have near zero probability of seasonal drought resiliency and were continuously unproductive and dry during late summer months.

Areas of forest, woodland, and agricultural crops within sagebrush landscapes were removed from the mesic grid using LANDFIRE and National Agricultural Statistics Service (NASS) croplands raster datasets (https://www.landfire.gov, https://www.nass.usda.gov). Datasets were summarized within the mesic grid and polygons containing individual or combined majority forest, woodland, or agricultural crop removed to eliminate sites uncorrelated to sage‐grouse habitats (Connelly, Rinkes, et al., 2011). Polygons containing alfalfa were retained due to sage‐grouse food resource values associated with this crop (Connelly, Rinkes, et al., 2011).

To reduce bias from mesic resources with low probability of sage‐grouse use, we quantified sagebrush cover proximal to individual mesic polygons by applying a neighborhood analysis using the LANDFIRE dataset. Polygons containing a cumulative sum of <10% sagebrush cover within a 1.0 km radius of their boundary were removed. This step eliminated center portions of large mesic complexes associated with irrigated hay and alfalfa fields. This procedure was not applied to the Great Plains due to rangeland composition containing lower shrub densities. The final mesic grid included approximately 600,000 polygons with probability greater than zero of productivity (i.e., NDVI ≥ 0.3) occurring during seasonal drought between 1984 and 2016.

Annual estimates of NPP were calculated by averaging Landsat images into single multispectral images for each of 32 years. Images were used to calculate seamless NDVI surfaces (30 × 30 m pixels) across the study area. Results provided a continuous measure of mean annual vegetative productivity during seasonal drought. The mesic grid was applied to each surface to calculate NDVI polygon means from intersected pixel values using zonal statistical functions. Final polygon summaries were representative of annual mesic productivity and patterns of ecological minimums that structure high value food resources for sage‐grouse.

### 2.3 Mesic resource types

Mesic resource polygons were categorized by types as “alfalfa,” “rangeland,” “riparian,” and “wet meadow,” to examine drought sensitivity and spatiotemporal patterns. Visual interpretation of high resolution imagery, in combination with decision support from ancillary spatial datasets, was used to classify polygons. Alfalfa sites were representative of irrigated cropland. In semi‐arid regions of the western United States, alfalfa is grown as a perennial crop that dominates agricultural production (Lindenmayer, Hansen, Brummer, & Pritchett, 2011). The NASS croplands data layer was used to automate identification of alfalfa.

Riparian and wet meadow areas were linked to shallow groundwater systems in floodplains that supported phreatophytic herbaceous and shrubland plant communities. We differentiated wet meadow and riparian sites within floodplains by confining riparian areas to vegetated corridors bordering stream channels. Wet meadows were visually discernible in aerial imagery due to dominance of herbaceous vegetation and land‐use practices associated with livestock pasture and hay production. The “rangeland” type was made up of upland herbaceous and shrubland plant communities. Rangeland sites inclusive to the mesic polygon grid exhibited lower sensitivity to seasonal drought and higher productivity relative to surrounding upland areas.

### 2.4 Linking mesic productivity to abiotic factors and land tenure

We estimated percent normal precipitation (PNP) annually (1984–2016) across the study area within a continuous 4 × 4 km grid by summing GRIDMET day of year precipitation data for each year and dividing it by the annual study period mean. All calculations were made within water years beginning 1 October and ending 30 September. Canadian Gridded Temperature and Precipitation Anomalies dataset (https://open.canada.ca) was used to augment Canadian regions not covered by GRIDMET. Gridded precipitation calculations were joined spatially to mesic polygons as attributes linking annual mesic productivity and PNP measures over 32 years. We estimated mesic polygon elevations by spatially joining the layer with 10 m resolution elevational data for the United States and Canada using a mean zonal statistical function. (https://nationalmap.gov, https://geogratis.gc.ca).

We estimated mesic sensitivity to seasonal drought intensity by plotting 32 years of NDVI measures against associated PNP values contained within the mesic polygon grid and fit linear regression models within and across individual mesic types to evaluate differences in sensitivity as represented by slope coefficients (*b*_1_). Estimates of the intercept (*b*_0_) can be interpreted as baseline productivity by region and mesic type given minimum PNP values. Separate plots were generated for Great Basin, Rocky Mountains, and Great Plains to examine response across gradients of ecosystem water balance. Data were plotted as point clouds due to high volume of points contained within each plot (*n* > 6 million). Clouds were fit with isobars to interpret shifts in point density relative to continuous PNP values.

Time series plots were used to examine trends in mesic site abundance relative to PNP from 1984 to 2016. Mesic resource abundance was calculated annually by summarizing mesic polygons (weighted by hectares) with NDVI values ≥0.3. All data summaries were stratified by region and mesic class. We estimated the influence of precipitation variance on mesic abundance trends by fitting linear regression models to annual mesic abundance sums (i.e., hectares of NDVI ≥ 0.3) and PNP variables. R‐squared values were calculated as an estimate of model fit.

Mean elevation estimates for productive mesic sites (NDVI ≥ 0.3) were calculated by type and region. Summaries were developed to identify potential altitudinal zonation patterns linked to orographic precipitation and snow retention (Litaor, Williams, & Seastedt, 2008). Density of mesic sites were plotted by elevation and linked to maps displaying their horizontal distribution within watersheds defined by USGS eight‐digit hydrologic units (https://nhd.usgs.gov). Watershed level results were provided as examples characteristic of broader mesic productivity patterns occurring during seasonal drought. For each watershed example, results were provided from first and third quartile PNP years, representing mesic availability at approximately 80% (Q_1_) and 107% (Q_3_) of mean annual precipitation for the period.

Proportional area of mesic resources was summarized by public and private land ownership. Ownership was assigned by intersecting the mesic polygon grid with GIS surface land ownership data for the western United States. (https://sagemap.wr.usgs.gov). Land ownership data for Canada were unavailable and not included in the summary.

### 2.5 Sage‐grouse

We examined availability of mesic sites to sage‐grouse during seasonal drought by summarizing its abundance proximal to known bird distributions. Sage‐grouse populations exhibit distinct clustering patterns that concentrate birds within the sagebrush biome (Doherty, Tack, Evans, & Naugle, 2010). Fitting data summaries to known bird distributions assured us that mesic patterns observed were characteristic of landscapes supporting sage‐grouse populations. Distributions were estimated by buffering all sage‐grouse lek locations (*n* = 6,304) by 10 km to account for observed spatial relationships among birds and mesic resources during seasonal drought (Donnelly et al., 2016). Only leks identified as active (male attendance >0) in the last 10 years were used. Lek locations are well documented for sage‐grouse and considered a reasonable index of bird distribution (Reese & Bowyer, 2007). Areas of Gunnison sage‐grouse were incorporated into estimates without spatial modification due to their limited distribution. Buffered lek areas encompassed 36 of 74 million hectares of current sage‐grouse range.

Within lek buffers, we calculated mesic resource density and distance between mesic sites relative to mean annual precipitation observed from 1984 to 2016 using only productive sites (NDVI ≥ 0.3). Distance between mesic sites was calculated by generating euclidean distance surfaces annually and estimating the mean for each year. Linear regression models were fit to individual regions to compare sensitivity of mesic density and distance to changing PNP.

Proportional abundance of sage‐grouse populations was estimated by region (Figure 1) using mean maximum male counts from 2015 lek surveys conducted within the project area. Lek surveys have been widely used by resource agencies to monitor trends in sage‐grouse populations and are considered an index of abundance (WAFWA, 2015).

### 2.6 Data processing

All image processing and raster‐based analyses, not otherwise noted, were conducted using Google Earth Engine cloud‐based geospatial processing platform (Gorelick et al., 2017). Vector‐based processing was completed using QGIS (qgis.osgeo.org). Plotting and statistical analyses were completed using the R Base Package (R Core Team, 2015).

## 3 RESULTS

Patterns of drought‐induced productivity were distinct within Great Basin, Rocky Mountains, and Great Plains. Landscape partitioning was representative of regional gradients in ecosystem water balance. Negatively correlated precipitation and PET confirmed dominance of cold season precipitation and hydrology influenced by high elevation snowpack in the Great Basin (*r* = −0.65) compared to summer rainfall‐driven events in the Great Plains. A low correlation (*r* = 0.04) in the Rocky Mountains indicated that precipitation was evenly distributed with a slight bias to cold season winter snowpack. Previous studies using ground‐based weather observation data (Lauenroth et al., 2014; Schlaepfer et al., 2012) corroborate spatial patterns derived from GRIDMET in this study.

**Figure 2:**
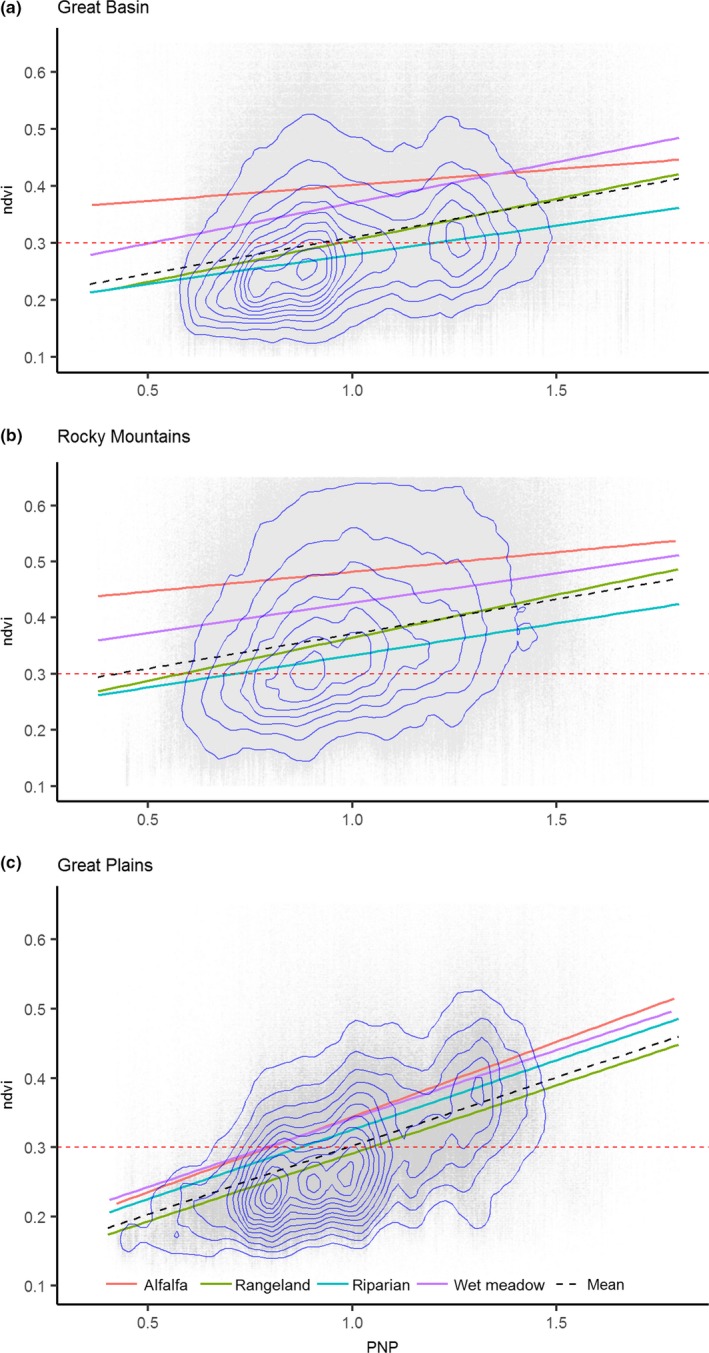
Sensitivity of mesic productivity (NDVI) to percent normal precipitation (PNP). Linear models fit to overall productivity mean and individually by mesic type. Blue isobars depict point cloud density where shorter distance between lines infers higher density of points. Horizontal dashed red line identifies threshold used to delineate productive (NDVI ≥ 0.3) and unproductive mesic sites (NDVI < 0.3)

Mesic productivity was 22% and 34% more sensitive to precipitation in the Great Plains (*b*_1_ = 2.00E‐03 NDVI/PNP) than the Great Basin (*b*_1_ = 1.64E‐03 NDVI/PNP) and Rocky Mountains (*b*_1_ = 1.49E‐03 NDVI/PNP; Figure 2). As a result, wet meadows and alfalfa were less sensitive to drought outside of the Great Plains (Figure 2a,b). Mesic sites were 52% and 97% more productive in the Rocky Mountains (Figure 2b, b0 = 0.203 NDVI) than the Great Basin (Figure 2a, b0 = 0.156 NDVI) and Great Plains (Figure 2c, b0 = 0.103 NDVI). Mesic rangeland sites were abundantly available to grouse in the Rocky Mountain region; outside this geography (Figure 2a,c), rangeland mesic sites were only productive (NDVI ≥ 0.3) in precipitation years at or above mean PNP.

**Figure 3:**
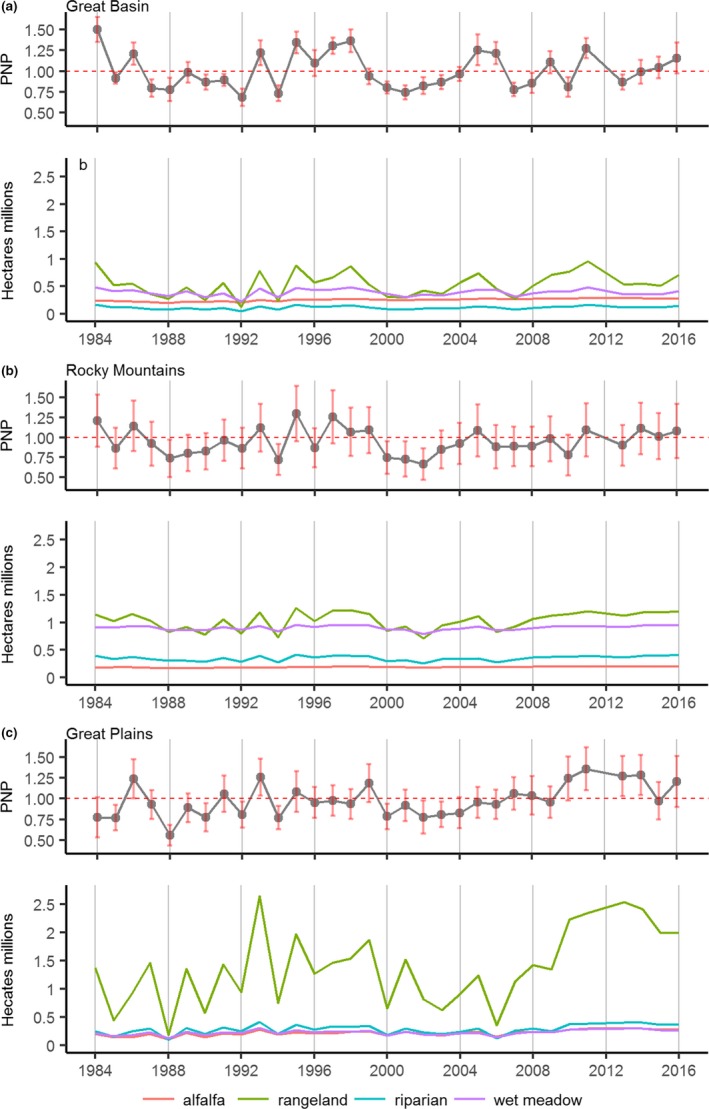
Yearly time series (1984–2016) of productive mesic sites (NDVI ≥ 0.3), binned by type and weighted by hectares. Plots paired with annual percent normal precipitation (PNP) trends for the Great Basin (a), Rocky Mountains (b), and the Great Plains (c)

Temporal variability in PNP over the past 33 years (Figure 3) explained ≥70% of mesic abundance in productive riparian (*R* ^2^ = 0.70, *p* < 2e‐16), wet meadow (*R* ^2^ = 0.70, *p* < 2e‐16), and rangeland (*R* ^2^ = 0.72, *p* < 2e‐16) sites, and ~50% of annual mesic abundance in alfalfa (*R* ^2^ = 0.47, *p* < 2.2e‐16). R‐squared statistics in individual regions were similar to overall values with the exception of Rocky Mountain alfalfa (*R* ^2^ = 0.23, *p* < 2e‐16), Great Basin riparian (*R* ^2^ = 0.78, *p* < 2e‐16) and wet meadow (*R* ^2^ = 0.77, *p* < 2e‐16) and Great Plains wet meadow (*R* ^2^ = 0.63, *p* < 2e‐16). Rangeland sites were most sensitive to drought, drying in response to PNP at rates six to nine times greater than riparian and wet meadow sites (Figure 3). Availability of productive rangelands varied most with PNP in the Great Plains (*b*_1_ = 63,108 ha/PNP) when compared to the Great Basin (*b*_1_ = 60,691 ha/PNP) and Rocky Mountains (*b*_1_ = 51,436 ha/PNP). Abundance of irrigated alfalfa showed the lowest overall rate of change relative to PNP (*b*_1_ = 3,577 ha/PNP).

**Figure 4:**
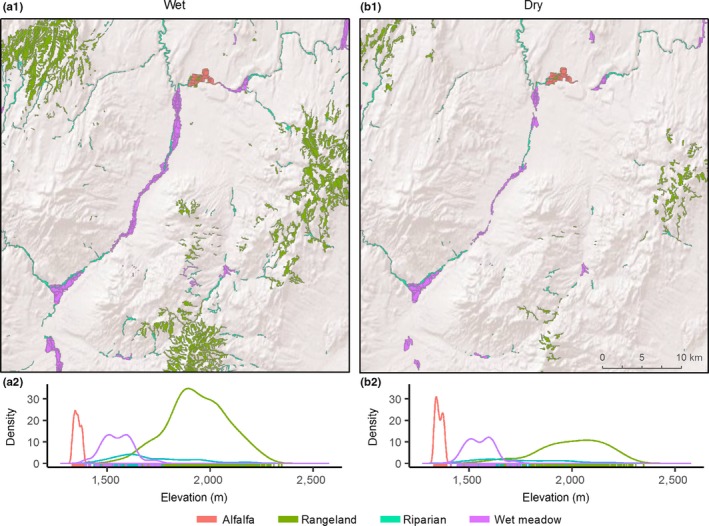
Great Basin example; Salmon Falls watershed displaying typical spatiotemporal shifts in productive mesic site (NDVI ≥ 0.3) abundance occurring during wet (a) and dry (b) years. Years are representative of third and first quartile annual precipitation measures occurring at 107% (a, Q_3_ = 2009) and 80% (b, Q_1_ = 2000) of annual percent normal precipitation from 1984–2016. Plots (a.2, b.2) show relative density of productive mesic sites by elevation during wet (a) and dry (b) years

**Figure 5:**
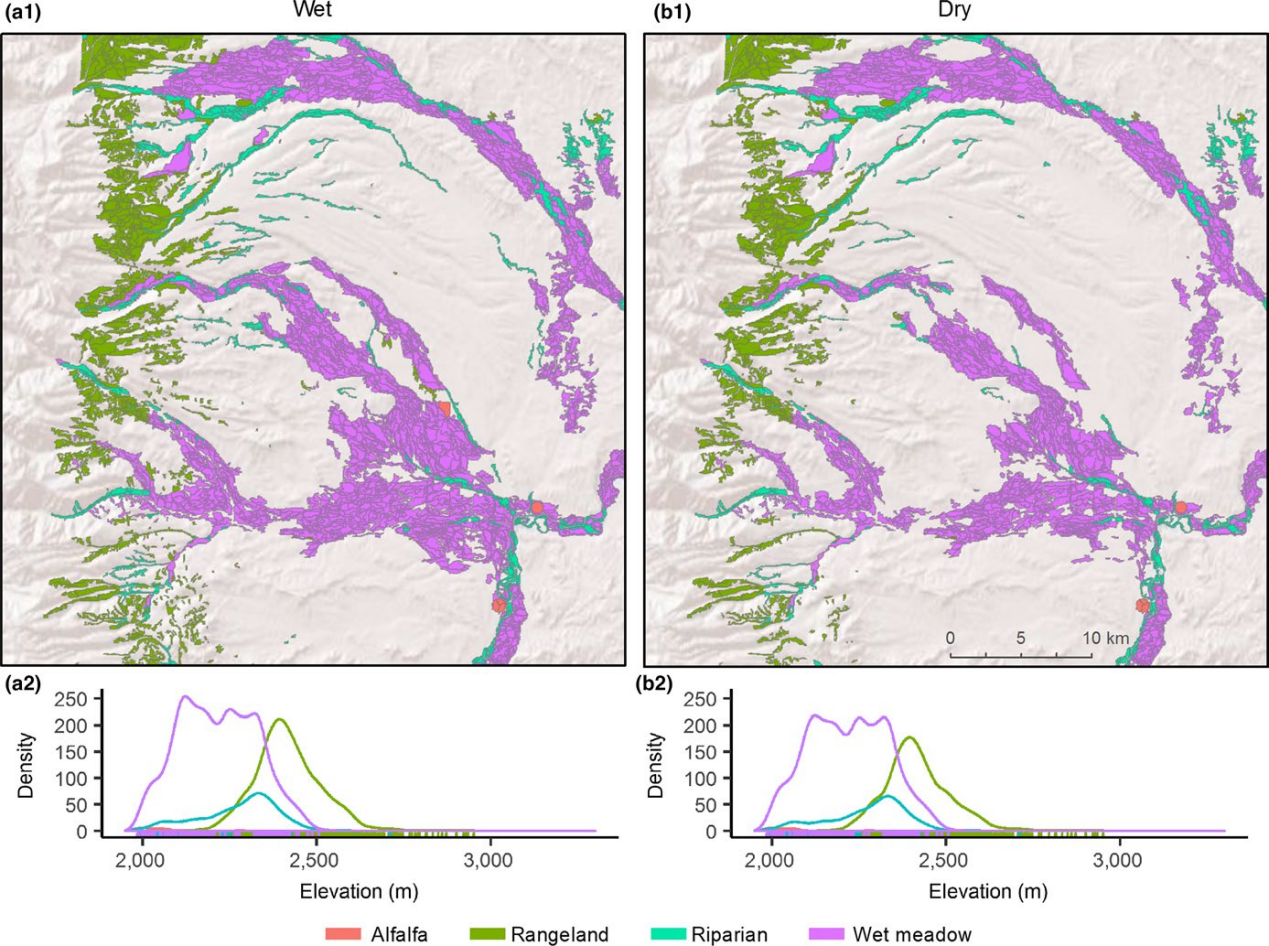
Rocky Mountains example; Upper Green River watershed displaying typical spatiotemporal shifts in high productivity mesic site (NDVI ≥ 0.3) abundance occurring during wet (a) and dry (b) years. Years are representative of third and first quartile annual precipitation measures occurring at 107% (a, Q_3_ = 2007) and 80% (b, Q_1_ = 1989) of annual percent normal precipitation from 1984–2016. Plots (a.2, b.2) show relative density of productive mesic sites by elevation during wet (a) and dry (b) years

**Figure 6:**
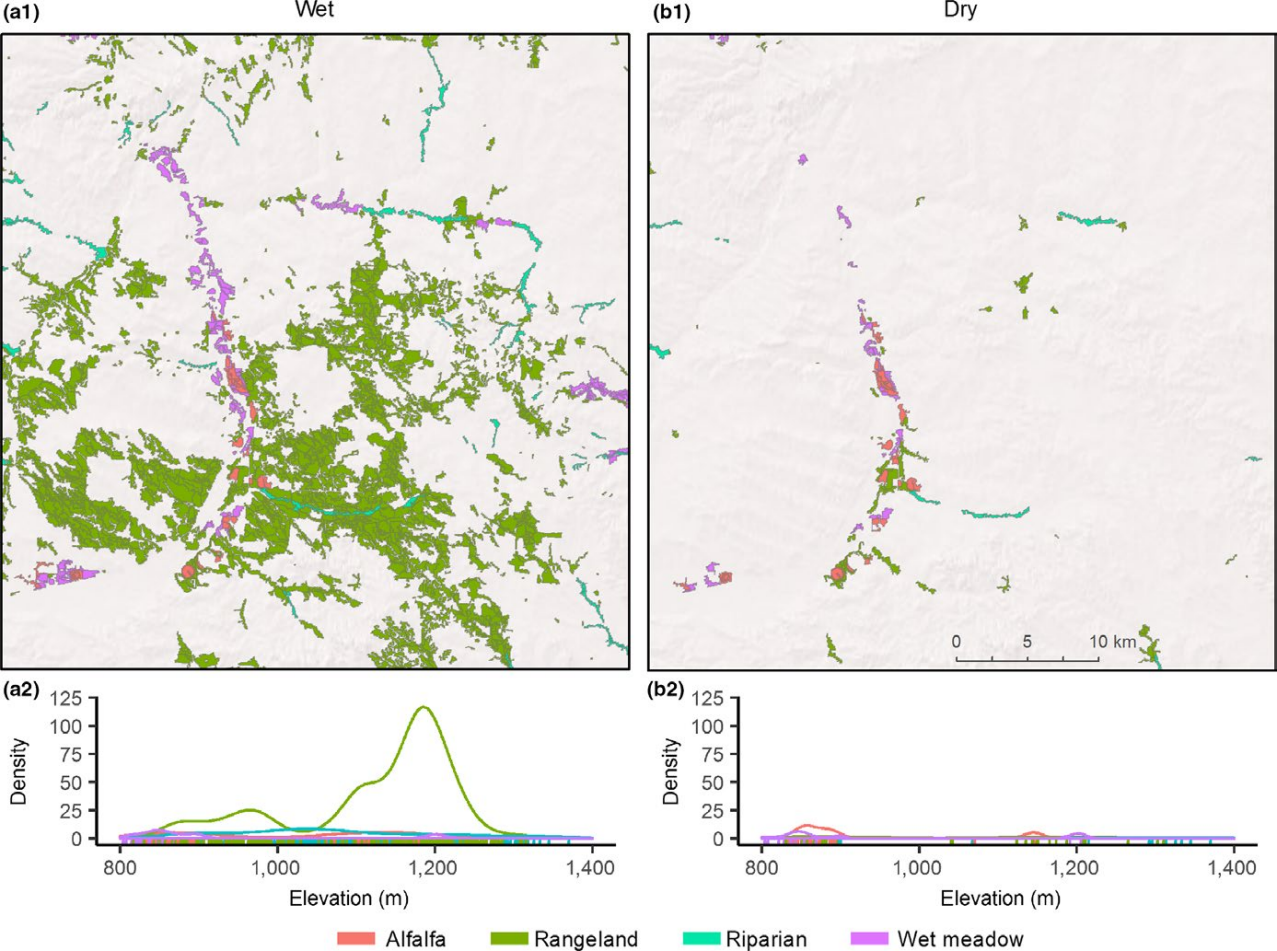
Great Plains example; Middle Musselshell watershed displaying typical spatiotemporal shifts in high productivity mesic site (NDVI ≥ 0.3) abundance occurring during wet(a) and dry(b) years. Years are representative of third and first quartile annual precipitation measures occurring at 106% (a, Q_3_ = 2011) and 80% (b, Q_1_ = 2001) of annual percent normal precipitation from 1984–2016. Plots (a.2, b.2) show relative density of productive mesic sites by elevation during wet (a) and dry (b) years

Drought sensitive rangelands occurred on average 420 and 339 m higher in watersheds than wet meadow systems in the Great Basin and Rocky Mountains (Table 1). These patterns were characteristic of elevational zonation associated with wet meadows and rangelands found in the regions (Figures 4 and 5). Least drought sensitive alfalfa occurred lower in watersheds in the Great Basin and Rocky Mountains and were 501 m and 643 m below rangeland sites (Table 1). Higher drought sensitivity in the Great Basin and the Great Plains dramatically altered the density and spatial distribution of productive mesic sites between above and below average precipitation years (Figures 4 and 6).

**Table 1:** Mean elevation (meters) of mesic type by region

Type	Great Basin	Rocky Mountains	Great Plains
Alfalfa	1,361	1,665	993
Rangeland	1,862	2,308	1,011
Riparian	1,573	2,014	1,042
Wet meadow	1,443	1,969	959

**Figure 7:**
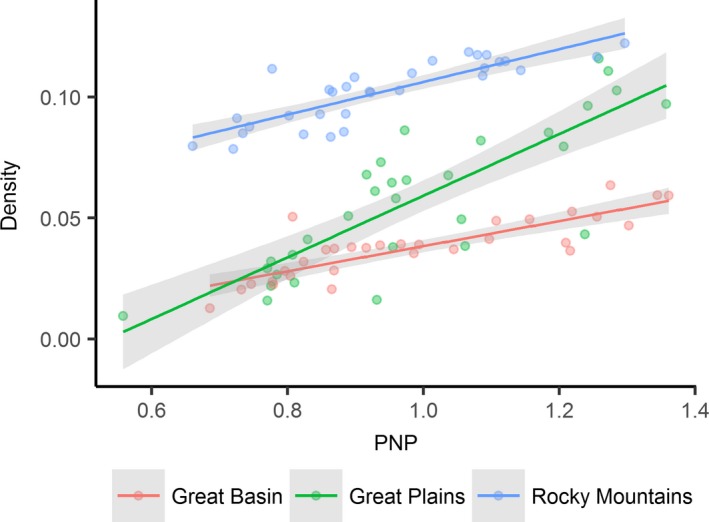
Average density of productive mesic sites relative to annual percent normal precipitation (PNP) for the period of study 1984–2016. Densities estimated within 10 km radius of known sage‐grouse lek locations (*n* = 6,304) partitioned by region

During seasonal drought, density of available mesic resources proximal to sage‐grouse distributions was five times higher in the Rocky Mountains (*b*_0_ = 0.038) than in the Great Basin (*b*_0_ = −0.013, Figure 7). Sensitivity of mesic density to changing PNP was approximately double in the Great Plains (*b*_1_ = 0.127 density/PNP) as opposed to the Great Basin (*b*_1_ = 0.051 density/PNP) and Rocky Mountains (*b*_1_ = 0.067 density/PNP). During low precipitation years, sage‐grouse populations in the Great Plains experienced limited mesic resource availability that was similar to the Great Basin; conversely, in high precipitation years, mesic resource abundance in the Great Plains climbed to levels equivalent to those characteristic of the Rocky Mountains (Figure 7).

**Figure 8:**
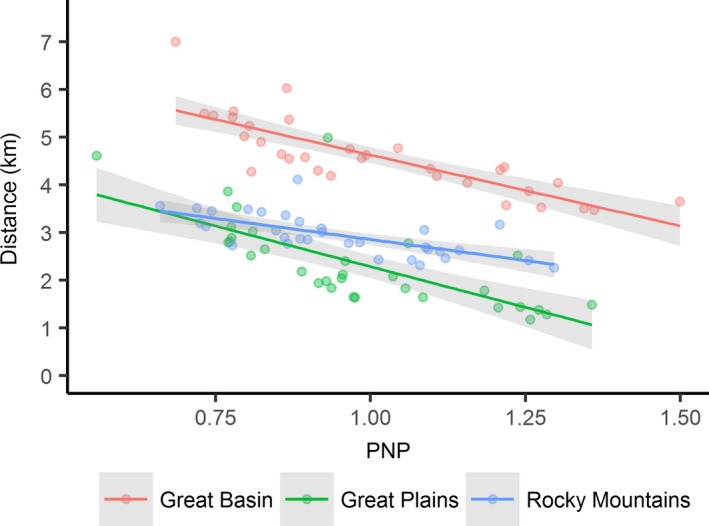
Average distance between productive mesic sites relative to annual percent normal precipitation (PNP) for the period of study 1984–2016. Distances estimated within 10 km radius of known sage‐grouse lek locations (*n* = 6,304) partitioned by region

Average distance between mesic sites in the Great Basin (4.6 km) was double that of the Great Plains (2.3 km) and 56% greater than in the Rocky Mountains (2.9 km). Maximum distances occurred in the driest years and were highest in the Great Basin (7.0 km) followed by the Great Plains (4.6 km) and Rocky Mountains (4.1 km, Figure 8). Mesic distance was nearly twice as sensitive to changing PNP in the Great Basin (*b_1_*=−3.0 km/PNP) and Great Plains (*b*_1_ = −3.3 km/PNP) versus the Rocky Mountains (*b*_1_ = −1.8 km/PNP, Figure 8) where the change in distance between wettest and driest years was 1.8 km as opposed to 3.5 km in other regions (Figure 8).

The largest proportion of the sage‐grouse population occurred in the Rocky Mountains (43%), followed by Great Basin (39%) and Great Plains (18%). Population density in the Rocky Mountains was 42% and 58% greater than the Great Basin and the Great Plains.

Within the sagebrush biome, private lands encompassed 40% of sage‐grouse range, but contained a disproportional 68% of mesic resources. Private ownership of mesic sites within regions averaged 59% (*SD *± 5%), 68% (*SD *± 4%), and 77% (*SD *± 3%) in the Great Basin, Rocky Mountains, and Great Plains, respectively. Nearly 90% of available wet meadow sites in all regions were privately owned; most productive rangelands were publicly owned in the Great Basin (66%) and Rocky Mountains (51%; Table 2). Riparian ownership was split evenly in the Great Basin with public lands containing a considerable proportion of sites in the Rocky Mountains (38%) and Great Plains (23%). Alfalfa ownership was 95%–97% private (Table 2).

**Table 2:** Proportion mesic ownership by type and region

Mesic type	Region	Private (%)	Public (%)
Alfalfa	Great Basin	97	3
	Rocky Mountains	97	3
	Great Plains	95	5
Rangeland	Great Basin	34	66
	Rocky Mountains	49	51
	Great Plains	76	24
Riparian	Great Basin	49	51
	Rocky Mountains	62	38
	Great Plains	77	23
Wet meadow	Great Basin	87	13
	Rocky Mountains	90	10
	Great Plains	86	14

## 4 DISCUSSION

Our analyses are the first to show that sage‐grouse populations are structured in part by biome‐wide gradients in ecosystem water balance that drive drought sensitivity in mesic resources (Figure 7). Correspondingly, landscapes with the greatest uncertainty in long‐term mesic availability supported the fewest birds. Documented shifts in mesic resource abundance across space and time were apparent (Figures 4–5) given climatic trends that reduced precipitation below three‐quarters of normal in 20% of years (1984–2016, Figure 3). Topography and seasonality of precipitation are primary mechanisms underpinning variation in density and distribution of mesic resources despite regional similarities in average annual precipitation (Lauenroth et al., 2014). At broad scales, spatial configuration of vegetative productivity is known to structure sage‐grouse distributions, with population abundance positively related to mesic availability (Donnelly et al., 2016). The causal linkage between rangewide bird densities and patterns of mesic resources we observed underscores the selective pressure of seasonal drought as an important determinant of landscape carrying capacity. Our regional findings provide the landscape context to previous studies suggesting weather‐driven productivity as a key factor influencing sage‐grouse survival (Blomberg et al., 2013; Guttery et al., 2013).

Through our findings we speculate that a diversity of mesic resources (i.e., rangelands, riparian, and wet meadows) rather than any single type is what sustains grouse populations over time. During drought, resilient wet meadows maintain adult survival as opposed to years of above average precipitation when resurgent mesic rangelands (Figures 4–5) maximize food availability and bird recruitment. Previous studies describe a slow‐paced life history of sage‐grouse (Connelly, Hagen, & Schroeder, 2011; Moynahan, Lindberg, & Thomas, 2006), recognizing that populations are reliant on relatively stable adult survival supplemented by occasional pulses in recruitment associated with favorable climatic conditions (Blomberg et al., 2012; Guttery et al., 2013). Regional drought sensitivity identified herein (Figure 2) may compound these relationships and act as ecological minimums to influence demographic performance differently across the species range. For example, in the Great Basin, distances between mesic sites were double in comparison to other regions (Figure 8) due to nonlinear patterns of intensifying drought that concentrated available mesic resources in wet meadow valley bottoms and high elevation rangelands (Figure 4), effectively extending the distance that young have to move between productive mesic sites to forage. Increased movements compound drought effects and are a factor known to lower brood survival (Gibson et al., 2017).

Our regional view of mesic resource constraints indicates that timing and intensity of rainfall events largely dictate bird response to increased mesic productivity in the Great Plains (Figures 2c and 6). Unlike climatic regimes in the Great Basin and Rocky Mountains, seasonal timing of precipitation in the Great Plains aligned with sage‐grouse nesting late March to mid‐June (Schroeder, Young, & Braun, 1999). Over the last 33 years, this region received half (52%) its annual precipitation in the nesting season period, which in typical years comes as short duration low intensity events. However, long duration intense rainfall events become more prevalent in wet years (Lauenroth & Bradford, 2009) with high mesic abundance (Figure 3c). Therefore, the right amount of precipitation in March‐June can enhance demographic performance (Blomberg et al., 2012); conversely, too much rain can reduce nest success (Moynahan, Lindberg, Rotella, & Thomas, 2007; Smith, 2016).

Sage‐grouse reliance on climatic intermittence explaining ≥70% of variance in mesic resource availability raises concerns over intensifying droughts predicted in the western United States (Dettinger, Udall, & Georgakakos, 2015). Our model depictions likely reflect a new normal in water scarcity that could compound impacts of demographic bottlenecks in Great Basin and Great Plains regions (Figures 4b and 6b). The immediate concern for sage‐grouse conservation is increased frequency and severity of droughts that influence ecological norms in semi‐arid regions (Trenberth, Dai, Rasmussen, & Parsons, 2003). Further complicating drought are altered fire regimes in some sagebrush ecosystems that may negate positive effects of precipitation and population growth (Coates et al., 2016). In the long‐term, climate projections that make systems more sensitive to drought could trigger mismatches in timing between resource availability and wildlife needs. Sage‐grouse have demonstrated some adaptive capacity to mitigate drought within existing climatic envelopes, but it is unlikely these traits portend projected landscape conditions (Gibson et al., 2017).

Our findings highlight the critical role of private lands in sage‐grouse conservation efforts in the western United States. As evidenced by 68% private ownership of mesic resources (Table 2). Significant conservation emphasis targets publicly managed uplands where birds mostly breed and nest (Doherty et al., 2010). Findings here support incentive‐based conservation efforts on private lands ([NRCS] Natural Resources Conservation Service, 2015) that ensure a holistic approach that includes drought resilient wet meadows (86%–90% private—see Table 2) important to supporting population maintenance during periods of water scarcity. Our data allow for new public‐private conservation strategies that account for regional variation in mesic resource ownership to promote cross‐boundary protection and restoration actions to help birds survive drought.

Sustainability of mesic resources hinges on maintenance of ecological processes and associated land‐use practices that foster drought resiliency (Gillson, Dawson, Jack, & McGeoch, 2013). Already being implemented are innovative techniques that restore degraded riparian systems by increasing mesic resistance to drought and elevating landscape productivity to benefit wildlife and ranching (Silverman et al., 2018). Another emerging solution is targeted removal of invasive conifer in high elevation sagebrush rangelands (Miller, Naugle, Maestas, Hagen, & Hall, 2017) known to increase snow retention and extend availability of soil water longer into the growing season (Kormos et al., 2017). Ecosystem benefits of invasive tree removal extend beyond sage‐grouse to include conservation of non‐target sagebrush‐obligate avifauna (Donnelly et al., 2017), enhancement of big game forage (Stephens, Johnston, Jonas, & Paschke, 2016), and promotion of ecosystem resilience to resistance to invasive species (Miller et al., 2017). To accelerate these efforts, we provide access to our mesic resource data through interactive web applications that allow users to track mesic productivity trends in sagebrush landscapes (Natural Resources Conservation Service‐Sage Grouse Initiative, 2017). Tools provide new perspective for private and public land managers by placing local conservation activities in the context of broad landscape functions that enable prioritization of protection and restoration actions of greatest ecological value.

## CONFLICT OF INTEREST

None declared.

## AUTHOR CONTRIBUTIONS

This study was conceived and designed by J.P.D. J.P.D., B.W.A., D.P., N.L.S., and J.D.T. analyzed the data. J.P.D., D.E.N., B.W.A., and V.J.D. obtained the resources. All other authors contributed to writing, reviewing, and editing the article. All authors approved of its submission.

## DATA ACCESSIBILITY

Data visualization and dynamic conservation tools created in partnership with Google are available from the Natural Resources Conservation Service via their Sage Grouse Initiative interactive map at map.sagegrouseinitiative.com.

## References

[ece34614-bib-0001] [MEAB] Millennium Ecosystem Assessment Board (2005). *Ecosystems and human well‐being: * *D* *esertification synthesis* . World Resources Institute. Retrieved from https://michebag.nl/handle/20.500.11822/955

[ece34614-bib-0002] [NRCS] Natural Resources Conservation Service (2015). Outcomes in conservation: Sage grouse initiative. Washington, DC: United States Department of Agriculture, Natural Resources Conservation Service.

[ece34614-bib-0003] Abatzoglou, J. T. (2013). Development of gridded surface meteorological data for ecological applications and modelling. International Journal of Climatology, 33(1), 121–131. 10.1002/joc.3413

[ece34614-bib-0004] Black, P. E. (2007). Revisiting the Thornthwaite and Mather Water Balance1. JAWRA Journal of the American Water Resources Association, 43(6), 1604–1605. 10.1111/j.1752-1688.2007.00132.x

[ece34614-bib-0005] Blomberg, E. J. , Sedinger, J. S. , Atamian, M. T. , & Nonne, D. V. (2012). Characteristics of climate and landscape disturbance influence the dynamics of greater sage‐grouse populations. Ecosphere, 3(6), 1–20. 10.1890/ES11-00304.1

[ece34614-bib-0006] Blomberg, E. J. , Sedinger, J. S. , Gibson, D. , Coates, P. S. , & Casazza, M. L. (2014). Carryover effects and climatic conditions influence the postfledging survival of greater sage‐grouse. Ecology and Evolution, 4(23), 4488–4499. 10.1002/ece3.1139 25512845 PMC4264898

[ece34614-bib-0007] Blomberg, E. J. , Sedinger, J. S. , Nonne, D. V. , & Atamian, M. T. (2013). Seasonal reproductive costs contribute to reduced survival of female greater sage‐grouse. Journal of Avian Biology, 44, 149–158. 10.1111/j.1600-048X.2012.00013.x

[ece34614-bib-0008] Bolger, D. T. , Patten, M. A. , & Bostock, D. C. (2005). Avian reproductive failure in response to an extreme climatic event. Oecologia, 142(3), 398–406. 10.1007/s00442-004-1734-9 15549403

[ece34614-bib-0009] Box, E. O. , Holben, B. N. , & Kalb, V. (1989). Accuracy of the AVHRR vegetation index as a predictor of biomass, primary productivity and net CO_2_ flux. Vegetatio, 80(2), 71–89. 10.1007/BF00048034

[ece34614-bib-0010] Coates, P. S. , Ricca, M. A. , Prochazka, B. G. , Brooks, M. L. , Doherty, K. E. , Kroger, T. , … Casazza, M. L. (2016). Wildfire, climate, and invasive grass interactions negatively impact an indicator species by reshaping sagebrush ecosystems. Proceedings of the National Academy of Sciences of the United States of America, 113(45), 12745–12750. 10.1073/pnas.1606898113 27791084 PMC5111658

[ece34614-bib-0011] Connelly, J. W. , Hagen, C. A. , & Schroeder, M. A. (2011). Characteristics and dynamics of greater sage‐grouse populations In Knick S. T., & Connelly J. W. (Eds.), Greater Sage‐Grouse: Ecology and conservation of a landscape species and its habitats (pp. 53–67). Berkeley, CA: University of California Press.

[ece34614-bib-0012] Connelly, J. W. , Rinkes, E. T. , & Braun, C. E. (2011). Characteristics of greater sage‐grouse habitats: A landscape species at micro and macro scales In Knick S. T., & Connelly J. W. (Eds.), Greater Sage‐Grouse: Ecology and conservation of a landscape species and its habitats (pp. 69–82). Berkeley, CA: University of California Press.

[ece34614-bib-0013] Dettinger, M. , Udall, B. , & Georgakakos, A. (2015). Western water and climate change. Ecological Applications: A Publication of the Ecological Society of America, 25(8), 2069–2093. 10.1890/15-0938.1 26910940

[ece34614-bib-0014] Doherty, K. E. , Tack, J. D. , Evans, J. S. , & Naugle, D. E. (2010). *Mapping breeding densities of greater sage‐grouse: a tool for range‐wide conservation planning* . United States Bureau of Land Management. Retrieved from https://www.conservationgateway.org/ConservationByGeography/NorthAmerica/UnitedStates/Documents/BLM-L10PG00911.pdf

[ece34614-bib-0015] Donnelly, J. P. , Naugle, D. E. , Hagen, C. A. , & Maestas, J. D. (2016). Public lands and private waters: Scarce mesic resources structure land tenure and sage‐grouse distributions. Ecosphere, 7(1), 1–15. 10.1002/ecs2.1208

[ece34614-bib-0016] Donnelly, J. P. , Tack, J. D. , Doherty, K. E. , Naugle, D. E. , Allred, B. W. , & Dreitz, V. J. (2017). Extending conifer removal and landscape protection strategies from sage‐grouse to songbirds, a range‐wide assessment. Rangeland Ecology & Management, 70(1), 95–105. 10.1016/j.rama.2016.10.009

[ece34614-bib-0017] Gibson, D. , Blomberg, E. J. , Atamian, M. T. , & Sedinger, J. S. (2017). Weather, habitat composition, and female behavior interact to modify offspring survival in Greater Sage‐Grouse. Ecological Applications: A Publication of the Ecological Society of America, 27(1), 168–181. 10.1002/eap.1427 28052504

[ece34614-bib-0018] Gilbert, N. (2011). United Nations considers creating advisory panel on land degradation akin to IPCC. Nature, 477, 262–264.21921893

[ece34614-bib-0019] Gillson, L. , Dawson, T. P. , Jack, S. , & McGeoch, M. A. (2013). Accommodating climate change contingencies in conservation strategy. Trends in Ecology & Evolution, 28(3), 135–142. 10.1016/j.tree.2012.10.008 23146578

[ece34614-bib-0020] Gorelick, N. , Hancher, M. , Dixon, M. , Ilyushchenko, S. , Thau, D. , & Moore, R. (2017). Google Earth engine: Planetary‐scale geospatial analysis for everyone. Remote Sensing of Environment, 202, 18–27. 10.1016/j.rse.2017.06.031

[ece34614-bib-0021] Guttery, M. R. , Dahlgren, D. K. , Messmer, T. A. , Connelly, J. W. , Reese, K. P. , Terletzky, P. A. , … Koons, D. N. (2013). Effects of landscape‐scale environmental variation on greater sage‐grouse chick survival. PLoS ONE, 8(6), e65582 10.1371/journal.pone.0065582 23824519 PMC3688806

[ece34614-bib-0022] Knapp, A. K. , & Smith, M. D. (2001). Variation among biomes in temporal dynamics of aboveground primary production. Science, 291(5503), 481–484. 11161201 10.1126/science.291.5503.481

[ece34614-bib-0023] Knick, S. T. , Hanser, S. E. , & Preston, K. L. (2013). Modeling ecological minimum requirements for distribution of greater sage‐grouse leks: Implications for population connectivity across their western range, USA. Ecology and Evolution, 3(6), 1539–1551. 10.1002/ece3.557 23789066 PMC3686190

[ece34614-bib-0024] Kormos, P. R. , Marks, D. , Pierson, F. B. , Williams, C. J. , Hardegree, S. P. , Havens, S. , … Svejcar, T. J. (2017). Ecosystem water availability in juniper versus sagebrush snow‐dominated rangelands. Rangeland Ecology & Management, 70(1), 116–128. 10.1016/j.rama.2016.05.003

[ece34614-bib-0025] Lauenroth, W. K. , & Bradford, J. B. (2009). Ecohydrology of dry regions of the United States: Precipitation pulses and intraseasonal drought. Ecohydrology, 2(2), 173–181. 10.1002/eco.53

[ece34614-bib-0026] Lauenroth, W. K. , Schlaepfer, D. R. , & Bradford, J. B. (2014). Ecohydrology of dry regions: Storage versus pulse soil water dynamics. Ecosystems, 17(8), 1469–1479. 10.1007/s10021-014-9808-y

[ece34614-bib-0027] Le Houérou, H. N. , Bingham, R. L. , & Skerbek, W. (1988). Relationship between the variability of primary production and the variability of annual precipitation in world arid lands. Journal of Arid Environments, 15(1), 1–18. 10.1016/S0140-1963(18)31001-2

[ece34614-bib-0028] Lindenmayer, R. B. , Hansen, N. C. , Brummer, J. , & Pritchett, J. G. (2011). Deficit irrigation of alfalfa for water‐savings in the Great Plains and intermountain. West: A Review and Analysis of the Literature, 103(1), 45 10.2134/agronj2010.0224

[ece34614-bib-0029] Litaor, M. I. , Williams, M. , & Seastedt, T. R. (2008). Topographic controls on snow distribution, soil moisture, and species diversity of herbaceous alpine vegetation, Niwot Ridge, Colorado. Journal of Geophysical Research, 113(G2), G02008 10.1029/2007JG000419

[ece34614-bib-0030] Loik, M. E. , Breshears, D. D. , Lauenroth, W. K. , & Belnap, J. (2004). A multi‐scale perspective of water pulses in dryland ecosystems: Climatology and ecohydrology of the western USA. Oecologia, 141(2), 269–281. 10.1007/s00442-004-1570-y 15138879

[ece34614-bib-0031] Maron, M. , McAlpine, C. A. , & Watson, J. (2015). Climate‐induced resource bottlenecks exacerbate species vulnerability: A review. Diversity, 21(21), 731–743. 10.1111/ddi.12339

[ece34614-bib-0032] Marvin, D. C. , Koh, L. P. , Lynam, A. J. , Wich, S. , Davies, A. B. , Krishnamurthy, R. , … Asner, G. P. (2016). Integrating technologies for scalable ecology and conservation. Global Ecology and Conservation, 7, 262–275. 10.1016/j.gecco.2016.07.002

[ece34614-bib-0033] Masek, J. G. , Vermote, E. F. , Saleous, N. E. , Wolfe, R. , Hall, F. G. , Huemmrich, K. F. , … Lim, T.‐K. (2006). A Landsat surface reflectance dataset for North America, 1990–2000. IEEE Geoscience and Remote Sensing Letters, 3(1), 68–72. 10.1109/LGRS.2005.857030

[ece34614-bib-0034] Melillo, J. M. , Richmond, T. T. , & Yohe, G. (2014). *Climate change impacts in the United States* . U.S. Global Change Research Program. Retrieved from https://admin.globalchange.gov/sites/globalchange/files/Ch_0a_FrontMatter_ThirdNCA_GovtReviewDraft_Nov_22_2013_clean.pdf

[ece34614-bib-0035] Miller, R. F. , Naugle, D. E. , Maestas, J. D. , Hagen, C. A. , & Hall, G. (2017). Special Issue: Targeted woodland removal to recover at‐risk grouse and their sagebrush‐steppe and prairie ecosystems. Rangeland Ecology & Management, 70(1), 1–8. 10.1016/j.rama.2016.10.004

[ece34614-bib-0036] Moynahan, B. J. , Lindberg, M. S. , Rotella, J. J. , & Thomas, J. W. (2007). Factors affecting nest survival of greater sage‐grouse in northcentral Montana. The Journal of Wildlife Management, 71(6), 1773–1783. 10.2193/2005-386

[ece34614-bib-0037] Moynahan, B. J. , Lindberg, M. S. , & Thomas, J. W. (2006). Factors contributing to process variance in annual survival of female greater sage‐grouse in Montana. Ecological Applications: A Publication of the Ecological Society of America, 16(4), 1529–1538. 16937816 10.1890/1051-0761(2006)016[1529:fctpvi]2.0.co;2

[ece34614-bib-0038] Natural Resources Conservation Service‐Sage Grouse Initiative (2017). *Mesic habitat conservation planning guide* . United States Department of Agriculture. Retrieved from https://www.sagegrouseinitiative.com/wp-content/uploads/2017/04/Mesic_Habitat_Conservation_Planning_Guide1.pdf

[ece34614-bib-0039] Noy‐Meir, I. (1973). Desert ecosystems: Environment and producers. Annual Review of Ecology and Systematics, 4, 25–51. 10.1146/annurev.es.04.110173.000325

[ece34614-bib-0040] Pastor, J. , Moen, R. , & Cohen, Y. (1997). Spatial heterogeneities, carrying capacity, and feedbacks in animal‐landscape interactions. Journal of Mammalogy, 78(4), 1040–1052. 10.2307/1383047

[ece34614-bib-0041] Pettorelli, N. , Vik, J. O. , Mysterud, A. , Gaillard, J.‐M. , Tucker, C. J. , & Stenseth, N. C. (2005). Using the satellite‐derived NDVI to assess ecological responses to environmental change. Trends in Ecology & Evolution, 20(9), 503–510. 10.1016/j.tree.2005.05.011 16701427

[ece34614-bib-0042] R Core Team (2015). R: A language and environment for statistical computing. Vienna, Austria: R Foundation for Statistical Computing.

[ece34614-bib-0043] Rajagopalan, B. , & Lall, U. (1998). Interannual variability in western US precipitation. Journal of Hydrology, 210(1), 51–67. 10.1016/S0022-1694(98)00184-X

[ece34614-bib-0044] Reese, K. P. , & Bowyer, R. T. (2007). Monitoring populations of sage‐grouse. Moscow, ID: University of Idaho, College of Natural Resources.

[ece34614-bib-0045] Rowland, M. M. , Wisdom, M. J. , Suring, L. H. , & Meinke, C. W. (2006). Greater sage‐grouse as an umbrella species for sagebrush‐associated vertebrates. Biological Conservation, 129(3), 323–335. 10.1016/j.biocon.2005.10.048

[ece34614-bib-0046] Sala, O. E. , Lauenroth, W. K. , & Golluscio, R. A. (1997). Semiarid plant functional types in temperate semiarid regions In Smith T. M., Shugaet H. H., & Woodward F. I. (Eds.), Plant functional types (pp. 217–233). Cambridge, UK: Cambridge University Press.

[ece34614-bib-0047] Sala, O. E. , Lauenroth, W. K. , & Parton, W. J. (1992). Long‐term soil water dynamics in the shortgrass steppe. Ecology, 73(4), 1175–1181. 10.2307/1940667

[ece34614-bib-0048] Schlaepfer, D. R. , Lauenroth, W. K. , & Bradford, J. B. (2012). Ecohydrological niche of sagebrush ecosystems. Ecohydrology, 5(4), 453–466. 10.1002/eco.238

[ece34614-bib-0049] Schlesinger, W. H. , Reynolds, J. F. , Cunningham, G. L. , Huenneke, L. F. , Jarrell, W. M. , Virginia, R. A. , & Whitford, W. G. (1990). Biological feedbacks in global desertification. Science, 247(4946), 1043–1048. 10.1126/science.247.4946.1043 17800060

[ece34614-bib-0050] Schroeder, M. A. , Aldridge, C. L. , Apa, A. D. , Bohne, J. R. , Braun, C. E. , Bunnell, S. D. , … Stiver, S. J. (2004). Distribution of Sage‐Grouse in North America. The Condor, 106(2), 363–376. 10.1650/7425

[ece34614-bib-0051] Schroeder, M. A. , Young, J. R. , & Braun, C. E. (1999). Greater Sage‐Grouse (*Centrocercus urophasianus*). The Birds of North America Online. Retrieved from https://birdsna.org/Species-Account/bna/species/saggro/introduction

[ece34614-bib-0052] Shirk, A. J. , Schroeder, M. A. , Robb, L. A. , & Cushman, S. A. (2017). Persistence of greater sage‐grouse in agricultural landscapes. The Journal of Wildlife Management, 81(5), 905–918. 10.1002/jwmg.21268

[ece34614-bib-0053] Silverman, N. L. , Allred, B. W. , Donnelly, J. P. , Chapman, T. B. , Maestas, J. D. , Wheaton, J. M. , … Naugle, D. E. (2018). Low‐tech riparian and wet meadow restoration increases vegetation productivity and resilience across semi‐arid rangelands. Restoration Ecology. 10.1111/rec.12869

[ece34614-bib-0054] Smith, J. T. (2016). *Landscape to local: A multi‐scale evaluation of voluntary efforts to reduce fragmentation and enhance management of rangelands for sage‐grouse* . PhD. University of Montana. Retrieved from https://search.proquest.com/openview/7c42d73558ceff1f869e6c4ac-564051d/1?pq-origsite=gscholar&cbl=18750&diss=y

[ece34614-bib-0055] Stephens, G. J. , Johnston, D. B. , Jonas, J. L. , & Paschke, M. W. (2016). Understory responses to mechanical treatment of Pinyon‐Juniper in Northwestern Colorado. Rangeland Ecology & Management, 69(5), 351–359. 10.1016/j.rama.2016.06.003

[ece34614-bib-0056] Trenberth, K. E. , Dai, A. , Rasmussen, R. M. , & Parsons, D. B. (2003). The changing character of precipitation. Bulletin of the American Meteorological Society, 84(9), 1205–1217. 10.1175/BAMS-84-9-1205

[ece34614-bib-0057] Vermote, E. , Justice, C. , Claverie, M. , & Franch, B. (2016). Preliminary analysis of the performance of the Landsat 8/OLI land surface reflectance product. Remote Sensing of Environment, 185, 46–56. 10.1016/j.rse.2016.04.008 PMC6999666 32020955

[ece34614-bib-0058] Vicente‐Serrano, S. M. , Gouveia, C. , Camarero, J. J. , Begueria, S. , Trigo, R. , Lopez‐Moreno, J. I. , … Sanchez‐Lorenzo, A. (2013). Response of vegetation to drought time‐scales across global land biomes. Proceedings of the National Academy of Sciences of the United States of America, 110(1), 52–57. 10.1073/pnas.1207068110 23248309 PMC3538253

[ece34614-bib-0059] [WAFWA ] Western Association of Fish and Wildlife Agencies (2015). *Greater sage‐grouse population trends ‐ an analysis of lek count databases 1965–2015* . Western Association of Fish and Wildlife Agencies. Retrieved from https://www.wafwa.org/Documents%20and%20Settings/37/Site%20Documents/News/Lek%20Trend%20Analysis%20final%208-14-15.pdf

[ece34614-bib-0060] Webb, W. L. , Lauenroth, W. K. , Szarek, S. R. , & Kinerson, R. S. (1983). Primary production and abiotic controls in forests, grasslands, and desert ecosystems in the United States. Ecology, 64(1), 134–151.

[ece34614-bib-0061] Weier, J. , & Herring, D. (2000). *Measuring vegetation (NDVI & EVI)* . Goddard Space Flight Center. Greenbelt, MD: NASA. Retrieved from https://earthobservatory.nasa.gov/Features/MeasuringVegetation/

[ece34614-bib-0062] Wenninger, E. J. , & Inouye, R. S. (2008). Insect community response to plant diversity and productivity in a sagebrush–steppe ecosystem. Journal of Arid Environments, 72(1), 24–33. 10.1016/j.jaridenv.2007.04.005

